# Development of a High-Throughput *Candida albicans* Biofilm Chip

**DOI:** 10.1371/journal.pone.0019036

**Published:** 2011-04-22

**Authors:** Anand Srinivasan, Priya Uppuluri, Jose Lopez-Ribot, Anand K. Ramasubramanian

**Affiliations:** 1 Department of Biomedical Engineering, The University of Texas at San Antonio, San Antonio, Texas, United States of America; 2 Department of Biology, The University of Texas at San Antonio, San Antonio, Texas, United States of America; 3 Department of South Texas Center for Emerging Infectious Diseases, The University of Texas at San Antonio, San Antonio, Texas, United States of America; Institute of Developmental Biology and Cancer Research, France

## Abstract

We have developed a high-density microarray platform consisting of nano-biofilms of *Candida albicans*. A robotic microarrayer was used to print yeast cells of *C. albicans* encapsulated in a collagen matrix at a volume as low as 50 nL onto surface-modified microscope slides. Upon incubation, the cells grow into fully formed “nano-biofilms”. The morphological and architectural complexity of these biofilms were evaluated by scanning electron and confocal scanning laser microscopy. The extent of biofilm formation was determined using a microarray scanner from changes in fluorescence intensities due to FUN 1 metabolic processing. This staining technique was also adapted for antifungal susceptibility testing, which demonstrated that, similar to regular biofilms, cells within the on-chip biofilms displayed elevated levels of resistance against antifungal agents (fluconazole and amphotericin B). Thus, results from structural analyses and antifungal susceptibility testing indicated that despite miniaturization, these biofilms display the typical phenotypic properties associated with the biofilm mode of growth. In its final format, the *C. albicans* biofilm chip (*Ca*BChip) is composed of 768 equivalent and spatially distinct nano-biofilms on a single slide; multiple chips can be printed and processed simultaneously. Compared to current methods for the formation of microbial biofilms, namely the 96-well microtiter plate model, this fungal biofilm chip has advantages in terms of miniaturization and automation, which combine to cut reagent use and analysis time, minimize labor intensive steps, and dramatically reduce assay costs. Such a chip should accelerate the antifungal drug discovery process by enabling rapid, convenient and inexpensive screening of hundreds-to-thousands of compounds simultaneously.

## Introduction

*Candida albicans* is the main causative agent of candidiasis, the most common fungal infection and now the third to fourth leading nosocomial infection in US hospitals [1], [2]. These infections have emerged as a growing threat to human health, especially for an increasing number of immunocompromised individuals who are at risk for opportunistic infections. The high mortality rate associated with candidiasis is in part due to the limited arsenal of antifungal drugs [3], [4], [5]. Another major reason is because candidiasis is frequently associated with biofilm formation on both inert and biological surfaces, and cells within these biofilms are intrinsically resistant to most antifungal agents and host defenses [6], [7]. Indeed, the increase in *Candida* infections in the last decades has almost directly paralleled the increase and widespread use of a broad range of medical implant devices [6], [7], [8], [9].

Biofilms are complex microbial communities, attached to a surface and typically encased within an exopolymeric material or matrix [10], [11], [12]. In the case of *C. albicans*, biofilms are composed of different morphological forms of the organism including yeast, pseudohyphae and hyphae, encased within an extracellular matrix [13], [14]. The antifungal resistance of biofilms is primarily attributed to increase in cell number and changes in genetic, physiological and molecular characteristics of the cells in the biofilm, and secondarily to slow diffusion of drugs, and/or binding of drugs to the biofilm matrix [15], [16], [17], [18], [19], [20]. Thus, there is a need to develop new strategies for the screening and discovery of antifungal drugs that prevent or control the formation of biofilms [21]. Although there are many different models for the formation of *C. albicans* biofilms *in vitro*; from the point of view of antifungal susceptibility testing, perhaps the most useful is the 96-well microtiter plate model [22], [23]. However, in the search for new and improved therapeutics, a field now dominated by high throughput screenings and hunger for speed, practical considerations of time, cost and efficiency severely limit the use of 96-well plate assays for probing diverse set of chemical libraries containing tens of thousands of molecules for new drugs; and novel, improved technologies are sorely needed.

Here we describe the development of a high-throughput microarray based technology for the formation of fungal biofilms. We present a *C. albicans* biofilm chip, or *Ca*BChip, that employs *C. albicans*, the main causative agent of candidiasis and the most common fungal infection. In its present format, *Ca*BChip consists of 768 spatially distinct and equivalent nano-biofilms, each with a volume of few tens of nanoliters, on a standard 1″×3″ glass slide. We expect these chips to accelerate the antifungal drug discovery process by enabling rapid, convenient and inexpensive screening of hundreds-to-thousands of compounds simultaneously.

## Materials and Methods

### Strain and culture conditions

*C. albicans* strain SC5314, a well characterized strain from the point of view of biofilm formation [22], [23], [24], was used throughout the study. Cells stored at −70°C as glycerol stocks were propagated by streaking a loopful of cells onto yeast peptone dextrose (YPD) agar (1% [wt/vol] yeast extract, 2% [wt/vol] peptone, 2% [wt/vol] dextrose) and incubated overnight at 37°C. A loopful of cells from YPD agar plates were inoculated into flasks (150 ml) containing 20 ml of YPD liquid media to be grown overnight in an orbital shaker (150–180 rpm) at 30°C. Under these conditions, *C. albicans* grows as budding-yeasts.

### Preparation of Functionalized Slides

Normal microscopic glass slides (1″×3″) (Fisher Scientific, Waltham, MA) were cleaned extensively to expose the silanol groups (-SiOH) on the surface. First, the slides were placed in a removable slide rack and washed by immersing them in a staining jar containing ethanol. The slides were then wiped clean using paper towels and air-dried using nitrogen gas. Next, the slide rack containing the slides was immersed in a dish filled with concentrated sulphuric acid and incubated for an overnight treatment. Finally, these slides were subjected to sonication and washed with Milli-Q water for 30 min, following another wash in acetone. This treatment exposed the silanol groups on the glass surface.

Clean slides were then coated with 3-aminopropyltriethoxysilane (APTES) (Sigma Aldrich, St. Louis, MO), by immersing the slide rack in APTES for 30 min. The slides were baked in the furnace at 110°C for 15 min. Baking allowed cross-linking of the APTES, resulting in glass slides with its surface expressing functional groups (-NH_2_-) of APTES. Finally the slides were spin coated with 1% (wt/vol in toluene) Polystyrene-Co-Maleic Anhydride (PS-MA) (Sigma) to achieve a mono-layer of hydrophobic coating.

### Optimization and printing of high density fungal cell arrays onto functionalized glass slides and subsequent biofilm development

A four-factor, two-level factorial design was performed using MINITAB (Minitab Solutions Inc., State College, PA) and DESIGN EXPERT (Stat-Ease Inc., Minneapolis, MN) software to obtain optimal conditions of media concentration, seeding density, collagen concentration and PSMA coating concentration for wash-resistant biofilm growth on the *Ca*BChip (Methods S1). The response (output) metrics were true biofilm yield and robust attachment, measured using microarray scanner and light microscope (Figure S1 and S2). For preparation of inocula for printing and biofilm formation in the *Ca*BChip, cells harvested from overnight YPD cultures were washed twice in sterile phosphate buffered saline (PBS; 10 mM phosphate buffer, 2.7 mM potassium chloride, 137 mM sodium chloride (pH 7.4) (Sigma) by centrifugation at 3000g. The cells were then resuspended in Reconstruction buffer (0.2N NaOH solution with 2.2% (wt/vol) Sodium Bicarbonate and 4.8% (wt/vol) HEPES). One hundred fold dilutions of the suspended cells were prepared and counted using a hemocytometer on a bright field microscope. Following count, a suspension of cells was prepared in reconstruction buffer at a cell density of 5×10^7^ cells/mL, which was diluted ten times by addition of 10× RPMI-1640 supplemented with L-glutamine and buffered with morpholinepropanesulfonic acid (Angus Buffers and Chemicals, Niagara Falls, NY) containing collagen (1.8 mg/ml) (Type 1 from rat tail, BD Biosciences, Bedford, MA), to give a final concentration of cells of 4×10^6^ cells/mL. The suspension containing yeast cells, collagen and media was printed (50 nL per spot) on the functionalized PSMA-coated glass slides using a microarray spotter (Omnigrid Micro, Digilab Inc., Holliston, MA). Printing was carried out by non-contact deposition using conically tapered 190 µm orifice ceramic tips (Digilab). The resulting spots were approximately 700 µm in diameter, spaced 1.2 mm apart; spots were printed in an array of 48 rows and 16 columns. In a standard print run, the tips were primed, rinsed in running water and vacuum dried twice after each loading and printing step, The cell suspension was kept on ice to prevent the gelation of collagen before printing and agitated gently just prior to placement on the printing robot to ensure a uniformly mixed cell suspension. A relative humidity of 97% was maintained during printing to prevent the drying of the biofilm spots. All microarray operations such as aspiration, dispensing, priming, printing and spatial distribution of array were controlled by AxSys program (Digilab). All surfaces, including the source plate station, wash and vacuum station, vacuum slide platter and printing chamber were sterilized by wiping with 70% isopropanol. In our experience, these procedures ensure that there is no detectable contamination of wells in the plates or of spots on the microarray. Immediately after printing, the slides were placed inside humidifier chambers (ArrayIt Corporation, Sunnyvale, CA) which were placed inside a 37°C incubator over different periods of time to allow for biofilm formation.

### Assessment of metabolic status of cells within biofilms

FUN 1 [2-chloro-4-(2,3-dihydro-3-methyl-(benzo-1,3-thiazol-2-yl)-methylidene)-1–phenylquinolinium iodide] (Invitrogen Corp., Carlsbad, CA) was used to stain and determine viability (levels of metabolic activity) of *C. albicans* cells within the biofilms formed in the chips. This membrane-permeable fluorescent dye is internalized and processed by metabolically active fungal cells, and has excitation and emission spectra that are compatible with the sets of lasers and filters installed in most microarray scanners. Briefly, the *Ca*BChip was stained with 0.5 µM FUN 1, by simply dunking the entire *Ca*BChip in a staining jar, and incubated in the dark at 37°C for 30 min. Following incubation, the chip was washed three times by dunking in PBS in order to remove excess stain. The slides were then air-dried and scanned in a microarray scanner (Genepix Personal 4100A, Axon Instruments, Union City, CA). A laser of 532 nm with a PMT gain of 270 was used to read the chip and fluorescent intensity of each spot was determined using GenePix 4.1 software (Axon). Fluorescence levels from FUN 1 staining were recorded as Relative Fluorescence Units (RFU). The microarray reader converts the real fluorescence signal into an electronic signal that can be “tuned” using the gain setting or sensitivity setting, and thus RFU is an arbitrary unit. Initial experiments indicated an excellent linear correlation between number of metabolically active cells spotted on the microarray and levels of FUN 1 fluorescence (Figure 4A).

### Microscopy techniques

Bright-field light microscopy techniques on an inverted microscope (Fisher Scientific) equipped for photography were used to routinely monitor biofilm formation, as a means of directly visualizing the overall morphology, distribution and topography of biofilms grown in the chip. The images were processed for display using Micron software (Westover Scientific, Bothell, WA). For scanning electron microscopy, biofilms formed in the chip were fixed with a solution of glutaraldehyde (2.5% w/v) in 0.1 M sodium cacodylate buffer at pH 7.4 for 2 h at 37°C. Following fixation the biofilms were treated with a solution of osmium tetraoxide (1% w/v) in 0.1 M sodium cacodylate buffer at pH 7.4 for 2 h at room temperature. The samples were rinsed with water and soaked in a series of ethanol solutions (a step gradient of 30%, 50%, 70%, and 90% in water for 10 min per step), ending with 100% ethanol. After dehydration, the samples were dried overnight in a vacuum dryer and subsequently coated with a 60∶40 gold-palladium alloy; approximately 10 nm thick using a Cressington Sputter coater for a duration of 30 sec. Scanning electron microscopy was performed using a Zeiss EVO 40 electron microscope (Carl Zeiss, Thronwood, NY). Confocal Scanning Laser Microscopy (CSLM) of FUN 1 stained biofilms to visualize three dimensional patterns and determine the architecture of the biofilms grown in the chip. CSLM was performed with a Zeiss LSM 510 confocal microscope (Carl Zeiss), using a rhodamine/fluorescein isothiocyanate protocol with excitation at 488 nm (argon ion laser). Images of sections in the *xy* plane were taken along the *z* axis, acquired by the resident software and processed using AutoQuant (Media Cybernetics, Bethesda, MD) and IMARIS 6.4 (Bitplane, St. Paul, MN).

### Susceptibility testing of cells within preformed *C. albicans* biofilms in CaBChip against antifungal agents

Susceptibility testing of cells within *C. albicans* biofilms in *Ca*BChip was performed against clinically used antifungal agents amphotericin B and fluconazole. Amphotericin B was obtained as a powder from Sigma (St. Louis, MO). A stock solution of Amphotericin B (1.6 mg/ml) was prepared in DMSO and stored at −20°C until used. Fluconazole was obtained as injection from Sicor Pharmaceuticals, Inc. (Irvine, CA). A stock solution of Fluconazole in 0.9% Sodium Chloride solution, available as injection was stored at 4°C until used. Subsequent dilutions of the antifungals were made in RPMI-1640 media supplemented with L-glutamine and buffered with MOPS. On top of the biofilms formed after 24 h, drugs of desired concentration of equal spot volume (50 nL), in two-fold dilutions were spotted using the robotic microarrayer. The *Ca*BChip(-s) containing drugs were then incubated in a humidified chamber for an additional 24 h, after which the slides were washed by gently dunking them in PBS. The *Ca*BChips were then stained with 0.5 µM FUN1 and the metabolic activity of cells within biofilms were read using the microarray scanner. Eight different concentrations of the drugs in six replicates, with appropriate positive (no drug) and negative (dead cells) controls were tested on a single *Ca*BChip. Multiple (at least two) chips were processed in parallel. Thus, depending upon the efficacy of the drug and its dose, each spot had different fluorescence levels and by quantifying these values at each spot, susceptibility profiles for each compound were determined. The fluorescence intensity of the control and dead (killed with sodium hypochlorite for 20 min) biofilms were arbitrarily set at 100% and 0% respectively, and the inhibitory effects of compounds were determined by the reduction in fluorescence intensity in relation to the controls, as measured in the microarray scanner. Data was calculated and expressed as percent biofilm inhibition relative to the average of the control wells. SMIC_50_ and SMIC_80_ values for each antifungal were determined as before [22], [23]. The calculated IC_50_ (Inhibitory Concentration of drugs required to reduce the fluorescence intensity by half, compared to live –controls) values were determined by fitting the variable slope Hill equation (an equation determining the non-linear drug dose-response relationship) using GraphPad Prism software (La Jolla, CA). For comparison purposes, antifungal susceptibility testing was also performed using the 96-well microtiter plate model of *C. albicans* biofilm formation previously developed by our group [22], [23], that uses the colorimetric (2,3-bis(2-methoxy-4-nitro-5-sulfo-phenyl)-2H-tetrazolium-5-carboxanilide) (XTT) assay as a measure of viability of cells within biofilms (see also Figure S3 and Table S1).

## Results and Discussion

Traditionally, most models for the formation of *C. albicans* biofilms are cumbersome, requiring expert handling, large volumes, long processing times and the use of specialized equipment not generally available in a regular microbiology laboratory. Frequently biofilms are grown on catheter disks or sheets placed inside a fermentor or a bioreactor under either static or dynamic flow conditions [14]. These culture techniques are slow, complex and demanding, and were mitigated to a great extent by the development of a 96-well microtiter plate model for the formation of *C. albicans* biofilms [22], [23]. In this model, fungal biofilms are formed on the bottom of the wells of microtiter plates, and the ability of metabolically active sessile cells to reduce a tetrazolium salt (XTT) to water-soluble orange formazan compounds, the intensity of which can then be determined using a microtiter-plate reader, is used as a semi-quantitative measurement of biofilm formation. This model was also adapted for antifungal susceptibility testing of cells within the biofilms [22], [23]. However, in this era dominated by high throughput demands and “hunger for speed”, the multiwell plate format still suffers from several limitations, most importantly inefficient liquid handling and removal without disturbing the biofilms, which severely limits the automation of this process. Other disadvantages of 96-well microtiter plate model include the need for relatively larger volume of reagents (thereby increasing costs) as well as incompatibility with high content experimentation and image analysis. A microarray format/platform ameliorates all these issues, thus allowing for *truly* high-throughput applications. Though the microarrays have been used to great benefit in the fields of genomics and proteomics, comparatively little effort has been directed toward using cellular microarrays, particularly in the case of pathogenic microorganisms [25], [26], [27], [28], [29], [30], [31].

### Design and Fabrication of the Candida albicans Biofilm Chip (CaBChip)

**Figure 1:**
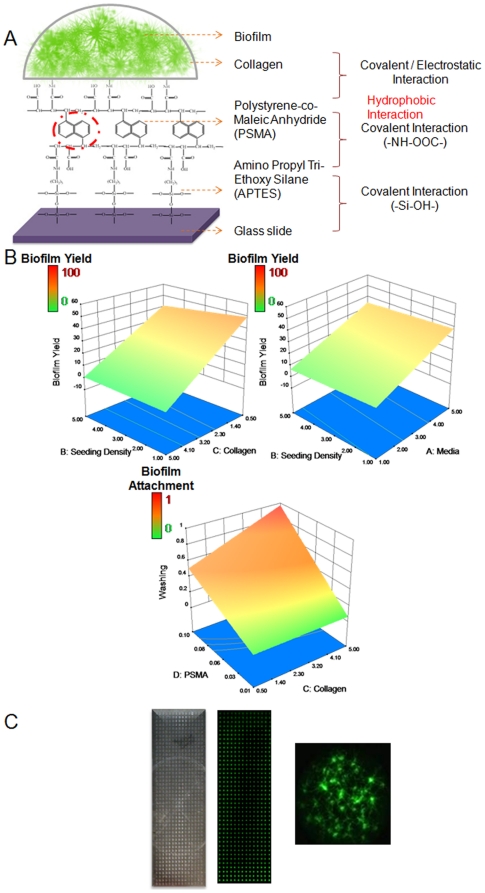
An overview of growth of fungal biofilms on glass substrates: preparation and characterization of the *C. albicans* biofilm chip. (**A**) Schematic of attachment of collagen-encapsulated *C. albicans* biofilms to modified glass slides. Glass slides modified with APTES were coated with PSMA and a suspension of *C. albicans* yeast cells in microbiological media and collagen as encapsulating material was spotted onto the glass slides. (**B**) The optimal concentrations of growth media, inocula, collagen and PSMA that supported maximum biofilm yield in spots that also attached robustly to substrate were obtained from a factorial design study. (**C**) A picture of the high-thoughput *Ca*BChip, printed using a robotic microarrayer and containing 768 spots on PSMA-coated slides. Each hemispherical spot is 50 nL in volume, approximately 700 µm in diameter, and with a 1.2 mm separation between spots. Also shown is a fluorescent picture of fungal biofilms formed on the chip and stained with FUN 1, taken with a microarray scanner, alongside a close-up image of nano-biofilms.

Our objective was to develop a high density microarray of spatially addressable three-dimensional biofilms of *C. albicans*, which should satisfy the following requirements: (i) firmly hold hundreds of spatially distinct biofilms on a single glass slide; (ii) forms a ‘true’ biofilm, and though small, displays phenotypic properties comparable to those of regularly-grown biofilms (*i.e.* growth, morphological and architectural characteristics and increased drug resistance); (iii) does not dry easily so that the cells may be cultured for prolonged periods of time; (iv) attaches robustly to the substrate and does not detach against multiple washings; and (v) fully compatible for analysis with a standard microarray scanner. We first performed a series of proof-of-concept experiments, mostly using manual pipetting prototypes, to check multiple parameters of biofilm formation, including those related to surface chemistry, matrix encapsulation, growth media and inocula preparation. The first requirement was that of a hydrophobic surface, allowing for discrete, independent liquid spots of small volume (in the nanoliters range) to be deposited on the surface of the microscope slide. Thus, the borosilicate (glass) slides were first pre-treated with 3-aminopropyltriethoxysilane (APTES), followed by coating with polystyrene-co-maleic anhydride (PSMA) (Figure 1A). The styrene-co-maleic anhydride molecules zip together forming a mono-layer made of two molecules in cross section, thus enhancing the hydrophobicity of the substrate [32]. The PSMA also provides sufficient functionality for subsequent binding to an encapsulating matrix such as collagen [31], [33]. The use of an encapsulating matrix was deemed necessary since initial experiments indicated that, in order to form robust (capable of withstanding the multiple washing steps) *C. albicans* biofilms on the microscope slides, it was required to encapsulate the fungal cells within a matrix. The use of collagen as the matrix of choice was mostly due to its optimal and easily controllable gelation characteristics, strong electrostatic/covalent binding to the functionalized surface, and the fact that it represents a biological substrate that mimics the tissue extracellular matrix *in vivo*. However, we also note that collagen encapsulation may limit the utility of the chip for the identification of compounds that affect early adherence or the biofilm matrix, as well as negatively influence the antifungal activity of compounds that bind protein with high avidity.

Next, using a two-level factorial design, we found the optimal operating parameters namely, concentrations of collagen, PSMA, *C. albicans* seeding density and growth media, that maximized the biofilm yield on spots that are stably attached to the substrate (Figure 1B). We observed that (i) high collagen concentration did not favor filamentation, and cells remained in planktonic form; and, on the other hand, low collagen concentration did not favor robust attachment of spots; (ii) excessively rich media resulted in hyper-filamentation forming a pseudo-biofilm, and poor media did not promote sufficient cell growth; (iii) high seeding density did not favor biofilm formation; and (iv) high PSMA coating concentration promoted stable attachment of spots. Hence, we concluded that a collagen concentration of 1.8 mg/ml with cell seeding density 4×10^6^ cells/ml in a 4× RPMI media printed on 0.1% PSMA-coated surfaces will yield most optimal biofilm chip (Figure S1 and S2). Finally, we printed the high density arrays using a robotic microarrayer. We robotically spotted a total of 768 spots of 50 nL each, containing a suspension of *C. albicans* yeast cells in collagen and microbiological media. This resulted in hemispherical spots that were 700 µm in diameter, with a spot-to-spot distance of 1.2 mm. After initial printing, the slides are simply incubated under inside humidifying chambers (to prevent drying) at 37°C to allow for biofilm development (Figure 1C). No additional media was added to the biofilm chip after initial spotting, which is in stark contrast with most models in which biofilms are submerged in large volumes of media. The extent of biofilm formation was assessed using FUN 1, a simple and sensitive assay for fluorescent staining of metabolically active fungal cells, which is fully compatible with standard microarray scanners.

### Growth characteristics and morphological and architectural features of *C. albicans* biofilms in the CaBChip

**Figure 2:**
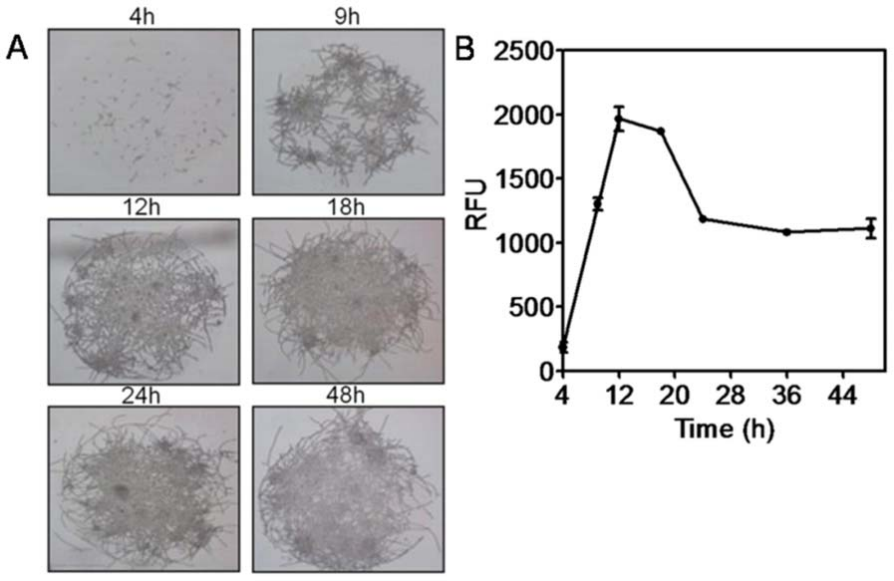
Kinetics of fungal biofilm formation on *Ca*BChip. (**A**) Light microscopy: a series of microphotographs showing the formation of nano-biofilms over time upon incubation at 37°C after initial printing using the robotic microarrayer. Magnification is ×100 for all panels. (**B**) Growth kinetics of *C. albicans* biofilm formation in *Ca*BChip as determined using FUN 1 staining and a microarray reader. Cells were printed on the chips, incubated at 37°C and fluorescence readings were taken over a 48 hour period. Results are expressed as arbitrary Relative Fluorescence Units (RFUs).

By using conventional methods for *C. albicans* biofilm formation, it is well established that the process of biofilm development occurs through different phases, including initial adherence of cells to a substrate, followed by growth and proliferation (which in the case of *C. albicans* is intimately associated with filamentation), and a final maturation phase that also includes accumulation of the extracellular matrix [13], [34]. To further establish the kinetics of biofilm formation in the chip format, we monitored the growth of viable cells in collagen gel spots on the *Ca*BChip over time using both microscopy and FUN 1 staining. This fluorescent dye is processed biochemically in the cytoplasm of living cells, forming cylindrical intravacuolar structures and rendering a fluorescent signal that can be read with a regular microarray reader using the appropriate excitation and emission filters. Similar to regular biofilms, direct bright-field microscopic observations revealed that *C. albicans* biofilms formed in the *Ca*BChip are composed of yeast, pseudohyphae and hyphae (Figure 2A). The results from these series of experiments indicated that, after a somewhat extended lag time of about 4 hours, cells grew rapidly and developed biofilms with maximum readings observed at approximately 12–18 h for what seem to be fully developed, complex biofilms. After 18 h, there was a reduction in the metabolic activity of biofilm cells, thus indicating that biofilms had reached maturity (Figure 2B). Thus, it would seem that, compared to other standard methods in which formation of mature biofilms typically occurs over 24–48 h and beyond [13], [34], [35], the process of biofilm development and maturation is somewhat accelerated in the chip, most likely due to the sub-microliter volume range, which in turn may result in faster nutrient depletion and accumulation of metabolic waste.

**Figure 3:**
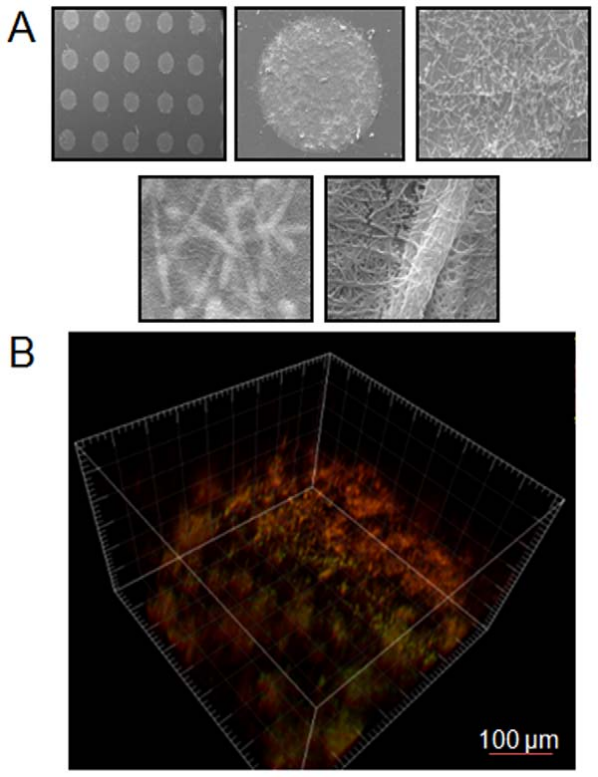
Characterization of morphological and architectural characteristics of fungal biofilms formed in *Ca*BChip. (**A**) A series of SEM images at increasing magnification (32×, 200×, 1.4k×, 8.2k× and 25.14k×) of *C. albicans* nano-films formed in *Ca*BChip after 24 h incubation. Hyphae enmeshed in collagen fibers can be clearly seen at the highest magnification. (**B**) Three-dimensional reconstruction of a FUN 1-stained *C. albicans* 24 h nano-biofilm formed in *Ca*BChip using CSLM and the associated software for the compilation of *xy* optical sections taken across the *z* axis.

In order to further ascertain the morphological and architectural characteristics of biofilms in the *Ca*BChip, we used SEM and CSLM. SEM provides a visual description of the biofilms at higher magnification. Shown in Figure 3A are a series of close-up images of spots in the biofilm chip with increasing magnification. At the highest magnification it can be seen that the fungal hyphae are embedded within the matrix of collagen fibers, which are approximately 2 µm and 100 nm in diameter, respectively. Contrary to SEM, the non-destructive nature of CSLM allows for the visualization of biofilms in its native state (Figure 3B). Results of FUN 1-stained biofilms using CSLM indicate that the biofilms formed in the *Ca*BChip show spatial heterogeneity, with regions of metabolically active cells interspersed within the extracellular matrix (composed of collagen as the encapsulating material and most likely also of exopolymeric material produced by biofilm cells), which is not stained by the metabolic dye. The thickness of the biofilm was estimated to be approximately 50 µm. Thus, from the point of view of their morphological, structural and architectural properties and despite several thousand-fold miniaturization, the nano-scale biofilms formed on the *Ca*BChip display phenotypic characteristics that are comparable to *C. albicans* biofilms formed using standard methodologies [34], [35].

### Validation of the CaBChip for high-throughput analyses and antifungal susceptibility testing

**Figure 4:**
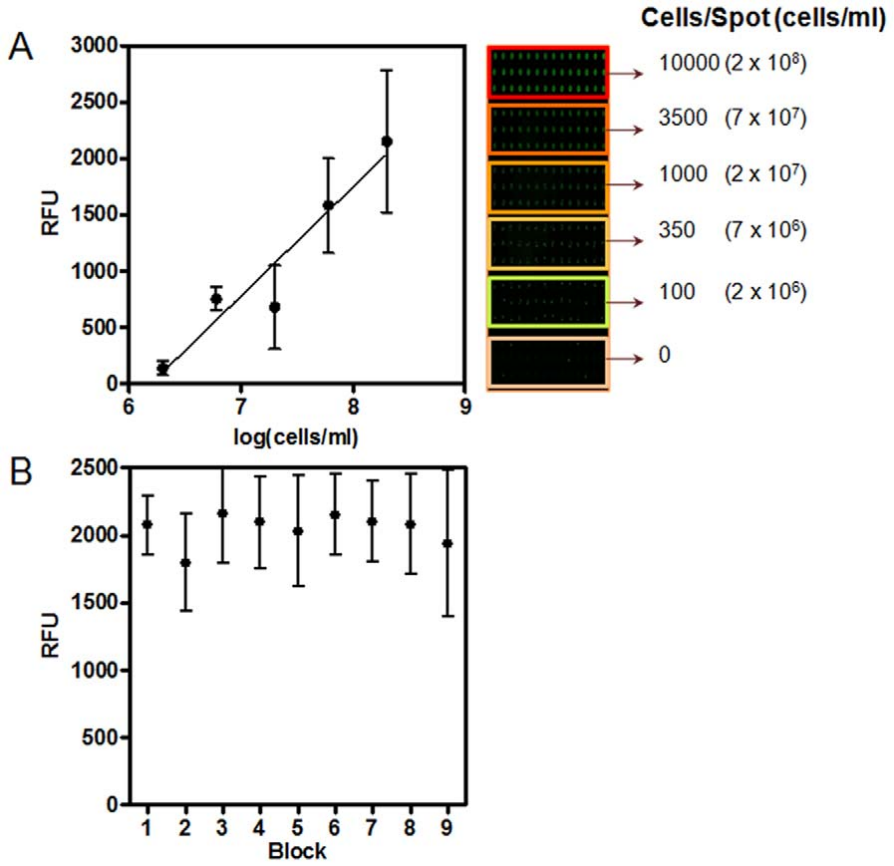
Validation of the fungal biofilm chip for high-throughput analyses. (**A**) Correlation of fluorescence intensities of spots obtained after FUN 1 staining with the number of viable cells present in each spot. The fluorescence intensities were measured using a microarray scanner and the values are expressed as RFUs. (**B**) Fluorescence intensity of nano-biofilms on *Ca*BChip stained with FUN 1. Values represent mean and standard deviations of multiple independent biofilms formed in each of 9 different blocks of the chip.

An important aspect of a high density array is the ability to make multiple measurements at a single time. Thus, it is imperative to demonstrate that all *C. albicans* biofilms formed in a same *Ca*BChip are equivalent to each other, which is essential for its future use in large scale high throughput/high content screening applications. To this end, we optimized the viability stain FUN 1 and operating parameters of the microarray scanner such that the fluorescence intensity correlated linearly with cell number over the range of interest (Figure 4A). We also found that the fluorescent intensities from spots that are seeded at same initial cell density were statistically indistinguishable (*P*<0.05, Figure 4B) indicating uniform distribution of biofilms at different locations on the *Ca*BChip. This demonstrates that the *Ca*BChip is a valid microarray-based platform for high-throughput screening techniques, including drug discovery or the screening of large collections of mutant strains, that will allow for the genetic dissection of the biofilm developmental process [36].

**Figure 5:**
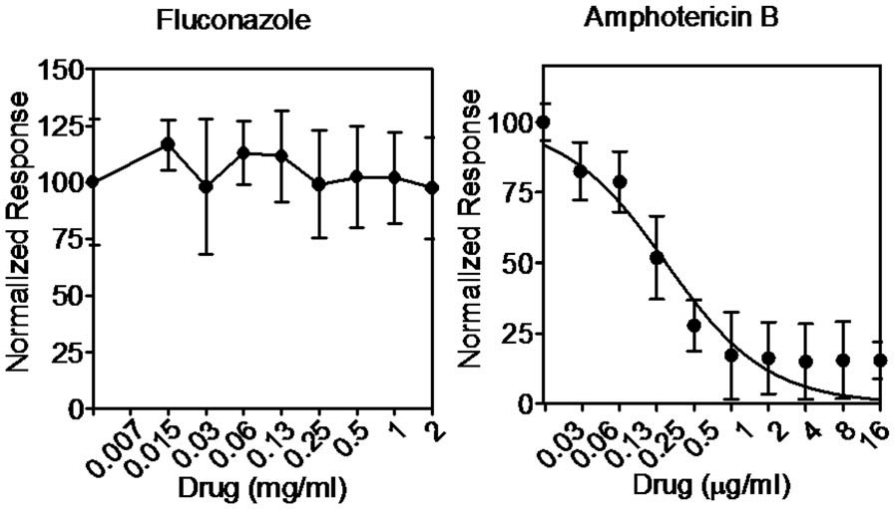
Dose-response curves of *Ca*BChip after treatment with antifungal drugs. Results of antifungal susceptibility testing and determination of IC_50_ values for fluconazole and amphotericin B using *Ca*BChip.

From a clinical perspective, one of the main negative consequences of biofilm formation is the high levels of antifungal drug resistance against most clinically used antifungal agents exhibited by *C. albicans* cells within biofilms [37]. This is one of the major contributors to the unacceptably high morbidity and mortality rates associated with candidiasis, despite of $3 billion per year spent on antifungal medications in the US alone [38]. Thus, there is an urgent need for the development of new and improved antifungal therapies, and the process of biofilm formation represents a very attractive target. In order to ascertain the functionality of *Ca*BChip in determining the susceptibility profiles of antifungal drugs against pre-formed biofilms, we carried out antifungal susceptibility testing of 24 h *C. albicans* biofilms grown on *Ca*BChip against fluconazole and amphotericin B. Using the robotic arrayer, drug concentrations of double-increments were spotted on top of the mature pre-formed biofilms on *Ca*BChip and incubated for an additional 24 h period, after which time FUN 1 was added. The fluorescence intensity of control (no drug) and sodium hypochlorite-treated dead biofilms were arbitrarily set at 100% and 0% respectively, and the inhibitory effects of the antifungal agents were determined by the reduction in fluorescence intensity in comparison to the controls. As in the case of biofilms formed using regular methods (*i.e.* 96 well microtiter plate model), we observed that the biofilms formed on *Ca*BChip were intrinsically resistant to fluconazole with SMIC_50_ and SMIC_80_ values of >1,024 µg/ml. From the corresponding curve (Figure 5), the calculated IC_50_ for fluconazole was >1,024 µg/ml. Since one of the main mechanisms of biofilm resistance against azole antifungal agents is binding of these compounds by beta-glucans in the extracellular matrix of the biofilm [15], [16], [17], [18], [19], [20], this provides additional evidence for the production of exopolymeric material by cells within the nanobiofilms. Also similar to regularly formed *C. albicans* biofilms, amphotericin B was effective against biofilms formed on *Ca*BChip, but only at relatively high concentrations. From the corresponding curve shown in Figure 5, the calculated IC_50_ for amphotericin B was 0.27±0.041 µg/ml, and the SMIC_50_ and SMIC_80_ values were 0.5 and 1 µg/ml, respectively. These results are consistent with those previously reported for biofilms formed using conventional techniques [22], [23], [34] (see also Figure S3 and Table S1) and once again further corroborate that, despite a near 2,000-fold miniaturization (compared to biofilms formed using the conventional 96-well microtiter plate model), the nanoscale biofilms on the *Ca*BChip display phenotypic properties, including high levels of antifungal drug resistance, that are similar to those formed using standard techniques. We note that preliminary assessment of echinocandin antifungals indicates low activity of these agents against the nanobiofilms (not shown). Compounds of this class are relatively highly protein bound [39] and it is possible that they bind to the encapsulating collagen matrix leading to decreased efficacy. We are further investigating these findings and testing a number of non-protein matrices for a second generation biofilm chip.

In summary, we have successfully developed a cell-based high density microarray, *Ca*BChip, for the formation of *C. albicans* nano-biofilms. Besides providing cell-specific islands on the substrate, the hydrophobic coating on the *Ca*BChip allows for an increased number of spots that could be printed in a microscope slide. In addition, the choice of a suitable hydrogel, as a “matrix” that encapsulates the individual biofilms, determines the robustness of the chip. Maintaining the integrity of the gel and controlling the adhesion of the matrix onto the substrate are critical for the robustness and performance of the chip. Despite nanoliter volume, the resulting biofilms demonstrate phenotypic characteristics that are consistent with the *C. albicans* biofilm mode of growth. The technology is flexible and we are currently adapting it to other fungal and bacterial organisms. Thus, the *Ca*BChip is truly high-throughput: it employs nano-scale cultures, enables rapid and easy handling, is amenable to automation and fully compatible with standard microarray technology and equipment. In its current format, a single *Ca*BChip replaces up to eight 96-well plates, and multiple chips can be printed and processed simultaneously. By virtue of its miniaturization and automation, the use of this technology platform minimizes manual labor, cuts reagents use and drastically reduces assay costs. By enabling rapid, convenient and inexpensive screening of hundreds-to-thousands of compounds simultaneously, the use of *Ca*BChip in high-content screening applications has the potential for changing the face of the antifungal drug discovery process.

## Supporting Information

Methods S1 **Factorial Designing of parameters for biofilm growth and attachment on biofilms.**
(DOCX)

Figure S1 **Influence of experimental parameters on biofilm yield and attachment.** (**A**) Light microscopy and FUN 1 staining shows that the biofilm yield is strongly dependent on media and collagen concentration, and weakly on PSMA coating concentration and initial seeding density; (**B**) Robust attachment of spots was dependent on PSMA coating and collagen concentration.
(TIF)

Figure S2 **Interaction plot of design variables.** Interaction plots show the influence of different variables on two observed outcomes: biofilm yield and robust attachment.
(TIF)

Figure S3 **Dose-response curves of 96 well-plate assay after treatment with antifungal drugs.** Determination of IC_50_ and SMIC_50_ and SMIC_80_ values for fluconazole and amphotericin B using the 96-well microtiter plate model of *C. albicans* biofilm formation, without (**A**) and with (**B**) collagen encapsulation.
(TIF)

Table S1 **IC_50_ in 96 well plate model.** The IC_50_ values are calculated from the dose-response curves of fluconazole and amphotericin B against biofilms formed in a 96 well plate.
(TIF)

## References

[1] Edmond MB, Wallace SE, McClish DK, Pfaller MA, Jones RN (1999). Nosocomial bloodstream infections in United States hospitals: a three-year analysis.. Clin Infect Dis.

[2] Pfaller MA, Diekema DJ (2007). Epidemiology of invasive candidiasis: a persistent public health problem.. Clin Microbiol Rev.

[3] Gudlaugsson O, Gillespie S, Lee K, Vande Berg J, Hu J (2003). Attributable mortality of nosocomial candidemia, revisited.. Clin Infect Dis.

[4] Viudes A, Peman J, Canton E, Ubeda P, Lopez-Ribot JL (2002). Candidemia at a tertiary-care hospital: epidemiology, treatment, clinical outcome and risk factors for death.. Eur J Clin Microbiol Infect Dis.

[5] Zaoutis TE, Argon J, Chu J, Berlin JA, Walsh TJ (2005). The epidemiology and attributable outcomes of candidemia in adults and children hospitalized in the United States: a propensity analysis.. Clin Infect Dis.

[6] Kojic EM, Darouiche RO (2004). Candida infections of medical devices.. Clin Microbiol Rev.

[7] Ramage G, Martinez JP, Lopez-Ribot JL (2006). Candida biofilms on implanted biomaterials: a clinically significant problem.. FEMS Yeast Res.

[8] Darouiche RO (2004). Treatment of infections associated with surgical implants.. N Engl J Med.

[9] Crump JA, Collignon PJ (2000). Intravascular catheter-associated infections.. Eur J Clin Microbiol Infect Dis.

[10] Bryers JD (2008). Medical biofilms.. Biotechnol Bioeng.

[11] Costerton JW, Cheng KJ, Geesey GG, Ladd TI, Nickel JC (1987). Bacterial biofilms in nature and disease.. Annu Rev Microbiol.

[12] Donlan RM (2002). Biofilms: microbial life on surfaces.. Emerg Infect Dis.

[13] Blankenship JR, Mitchell AP (2006). How to build a biofilm: a fungal perspective.. Curr Opin Microbiol.

[14] Ramage G, Saville SP, Thomas DP, Lopez-Ribot JL (2005). Candida biofilms: an update.. Eukaryot Cell.

[15] Jabra-Rizk MA, Falkler WA, Meiller TF (2004). Fungal biofilms and drug resistance.. Emerg Infect Dis.

[16] Nett J, Lincoln L, Marchillo K, Massey R, Holoyda K (2007). Putative role of beta-1,3 glucans in Candida albicans biofilm resistance.. Antimicrob Agents Chemother.

[17] Nett JE, Crawford K, Marchillo K, Andes DR (2010). Role of Fks1p and matrix glucan in Candida albicans biofilm resistance to an echinocandin, pyrimidine, and polyene.. Antimicrob Agents Chemother.

[18] Nett JE, Sanchez H, Cain MT, Andes DR (2010). Genetic basis of Candida biofilm resistance due to drug-sequestering matrix glucan.. J Infect Dis.

[19] Perumal P, Mekala S, Chaffin WL (2007). Role for cell density in antifungal drug resistance in Candida albicans biofilms.. Antimicrob Agents Chemother.

[20] Ramage G, Bachmann S, Patterson TF, Wickes BL, Lopez-Ribot JL (2002). Investigation of multidrug efflux pumps in relation to fluconazole resistance in Candida albicans biofilms.. J Antimicrob Chemother.

[21] Ramage G, Mowat E, Jones B, Williams C, Lopez-Ribot J (2009). Our current understanding of fungal biofilms.. Crit Rev Microbiol.

[22] Pierce CG, Uppuluri P, Tristan AR, Wormley FL, Mowat E (2008). A simple and reproducible 96-well plate-based method for the formation of fungal biofilms and its application to antifungal susceptibility testing.. Nat Protoc.

[23] Ramage G, Vande Walle K, Wickes BL, Lopez-Ribot JL (2001). Standardized method for in vitro antifungal susceptibility testing of Candida albicans biofilms.. Antimicrob Agents Chemother.

[24] Uppuluri P, Chaturvedi AK, Srinivasan A, Banerjee M, Ramasubramaniam AK (2010). Dispersion as an important step in the Candida albicans biofilm developmental cycle.. PLoS Pathog.

[25] Fesenko DO, Nasedkina TV, Prokopenko DV, Mirzabekov AD (2005). Biosensing and monitoring of cell populations using the hydrogel bacterial microchip.. Biosens Bioelectron.

[26] Gefen O, Balaban NQ (2008). The Moore's Law of microbiology - towards bacterial culture miniaturization with the micro-Petri chip.. Trends Biotechnol.

[27] Groisman A, Lobo C, Cho H, Campbell JK, Dufour YS (2005). A microfluidic chemostat for experiments with bacterial and yeast cells.. Nat Methods.

[28] Hart T, Zhao A, Garg A, Bolusani S, Marcotte EM (2009). Human cell chips: adapting DNA microarray spotting technology to cell-based imaging assays.. PLoS One.

[29] Ingham CJ, Sprenkels A, Bomer J, Molenaar D, van den Berg A (2007). The micro-Petri dish, a million-well growth chip for the culture and high-throughput screening of microorganisms.. Proc Natl Acad Sci U S A.

[30] Narayanaswamy R, Niu W, Scouras AD, Hart GT, Davies J (2006). Systematic profiling of cellular phenotypes with spotted cell microarrays reveals mating-pheromone response genes.. Genome Biol.

[31] Lee MY, Kumar RA, Sukumaran SM, Hogg MG, Clark DS (2008). Three-dimensional cellular microarray for high-throughput toxicology assays.. Proc Natl Acad Sci U S A.

[32] Garnier G, Duskova-Smrckova M, Vyhnalkova R, van de Ven TGM, Revol JF (2000). Association in solution and adsorption at an air-water interface of alternating copolymers of maleic anhydride and styrene.. Langmuir.

[33] Ivanova EP, Pham DK, Demyashev GM, Nicolau DV (2002). Oligonucleotide/poly(l-lysine) complexes attachment on poly(styrene/maleic acid) and poly(styrene/maleic anhydride polymeric surfaces.. Proceedings of SPIE.

[34] Chandra J, Kuhn DM, Mukherjee PK, Hoyer LL, McCormick T (2001). Biofilm formation by the fungal pathogen Candida albicans: Development, architecture, and drug resistance.. Journal of Bacteriology.

[35] Ramage G, Vandewalle K, Wickes BL, Lopez-Ribot JL (2001). Characteristics of biofilm formation by Candida albicans.. Rev Iberoam Micol.

[36] Nobile CJ, Mitchell AP (2006). Genetics and genomics of Candida albicans biofilm formation.. Cell Microbiol.

[37] Uppuluri P, Pierce CG, Lopez-Ribot JL (2009). Candida albicans biofilm formation and its clinical consequences.. Future Microbiology.

[38] Wilson LS, Reyes CM, Stolpman M, Speckman J, Allen K (2002). The direct cost and incidence of systemic fungal infections.. Value Health.

[39] Odabasi Z, Paetznick V, Rex JH, O-Z L (2007). Effects of serum on in vitro susceptibility testing of echinocandins.. Antimicrob Agents Chemother.

