# Supplementary material for: Development of a High-Throughput Candida albicans Biofilm Chip
Source: PLoS One. 2011 Apr 22;6(4):e19036. doi: 10.1371/journal.pone.0019036 (PMC3081316; doi:10.1371/journal.pone.0019036)
Supplement: Methods S1 — Factorial Designing of parameters for biofilm growth and attachment on biofilms. (DOCX) [file pone.0019036.s001.docx]

The values for the four independent variables, media, seeding density, collagen and PSMA concentration are shown in Figure S1. The levels of the factors were chosen based on a combination of preliminary experimental and literature data. Sixteen (2^4^) different combinations of independent variables, and their levels were tested in duplicate on the *Ca*BChip. The order of the experiments, wherein each level of a factor was combined with other factors once, was generated randomly in order to minimize systematic error (MINITAB). The effect of these factors on the design was ascertained from the response variables, namely, biofilm yield and attachment. Biofilm yield was scaled between 0 – 100% based on the formation of ‘true’ biofilm with high cell mass, and attachment was given a heuristic metric of 0, 0.5 or 1 for low, medium and high attachment, respectively.
