# Supplementary figures and images for: Development of a High-Throughput Candida albicans Biofilm Chip
Source: PLoS One. 2011 Apr 22;6(4):e19036. doi: 10.1371/journal.pone.0019036 (PMC3081316; doi:10.1371/journal.pone.0019036)

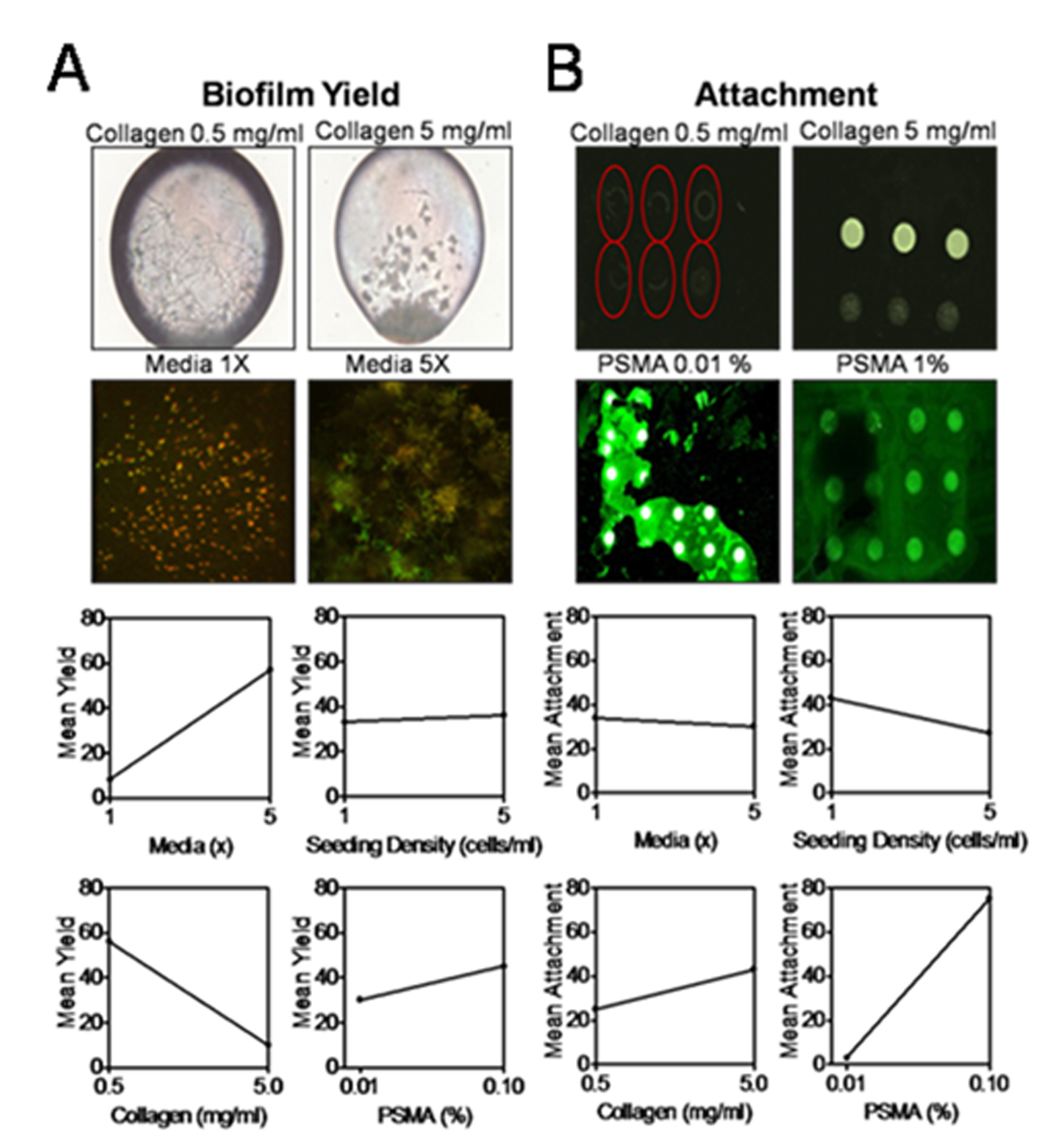

Supplement: Figure S1 — Influence of experimental parameters on biofilm yield and attachment. (A) Light microscopy and FUN 1 staining shows that the biofilm yield is strongly dependent on media and collagen concentration, and weakly on PSMA coating concentration and initial seeding density; (B) Robust attachment of spots was dependent on PSMA coating and collagen concentration. (TIF) [file pone.0019036.s002.tif]

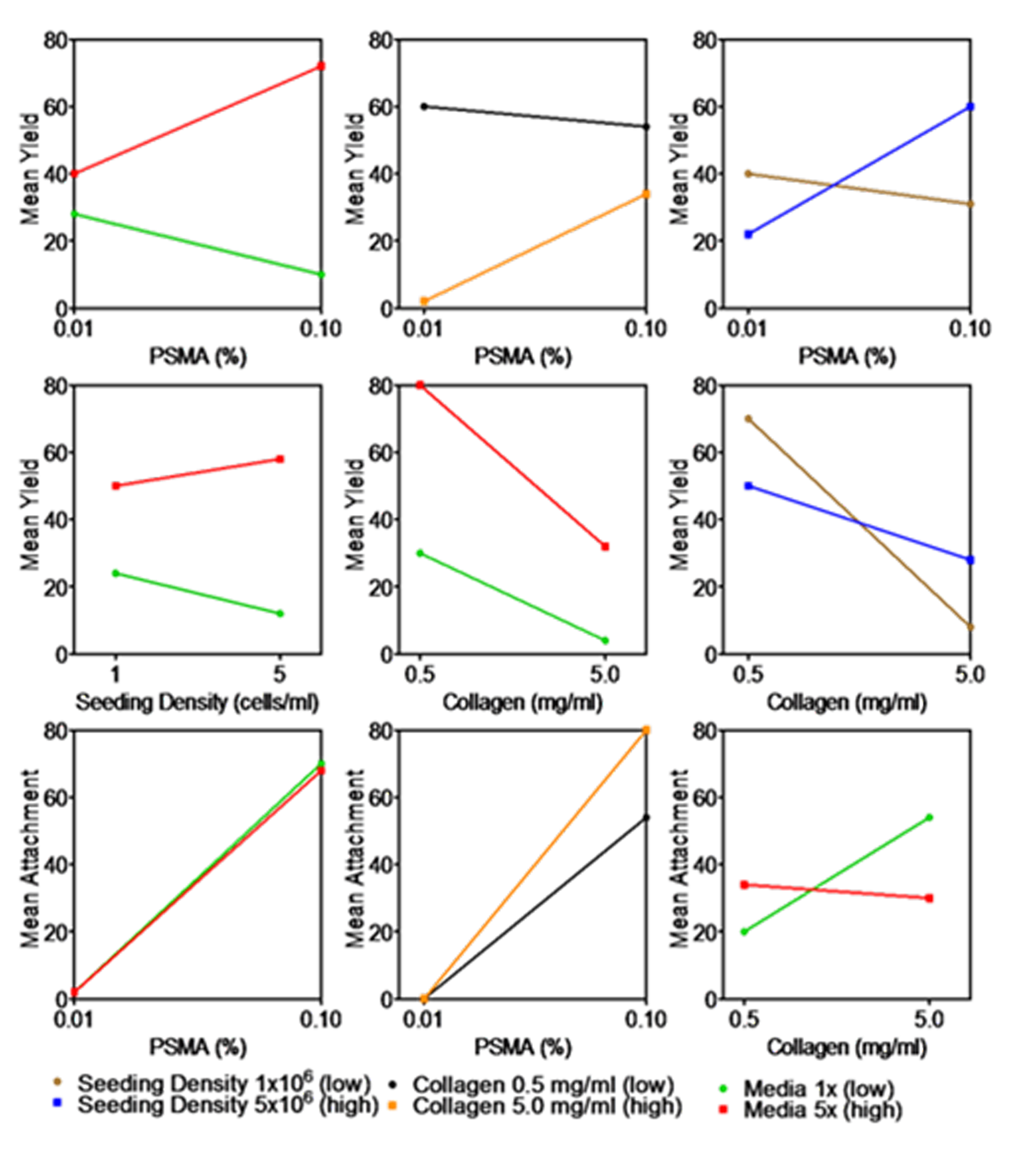

Supplement: Figure S2 — Interaction plot of design variables. Interaction plots show the influence of different variables on two observed outcomes: biofilm yield and robust attachment. (TIF) [file pone.0019036.s003.tif]

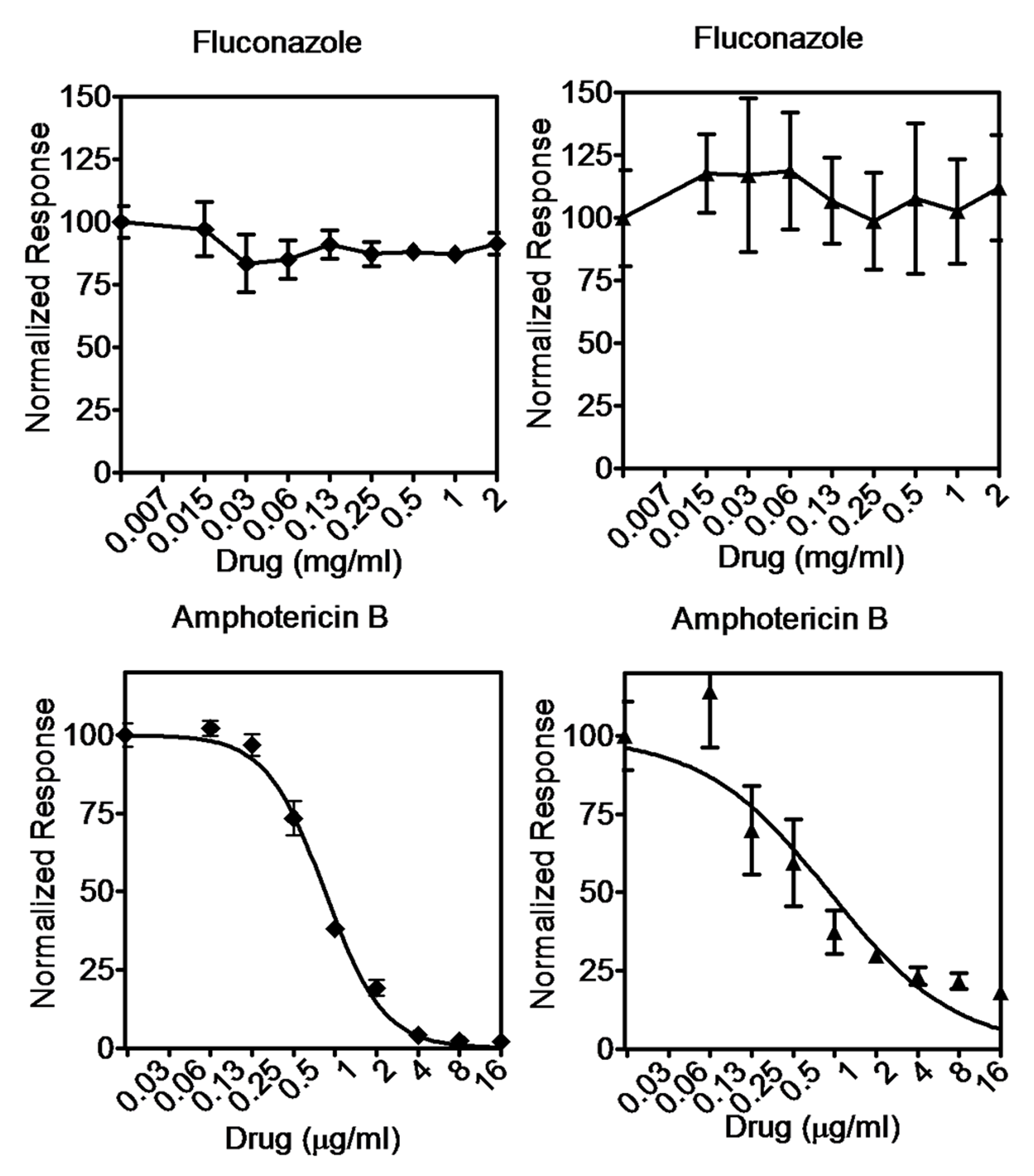

Supplement: Figure S3 — Dose-response curves of 96 well-plate assay after treatment with antifungal drugs. Determination of IC50 and SMIC50 and SMIC80 values for fluconazole and amphotericin B using the 96-well microtiter plate model of C. albicans biofilm formation, without (A) and with (B) collagen encapsulation. (TIF) [file pone.0019036.s004.tif]

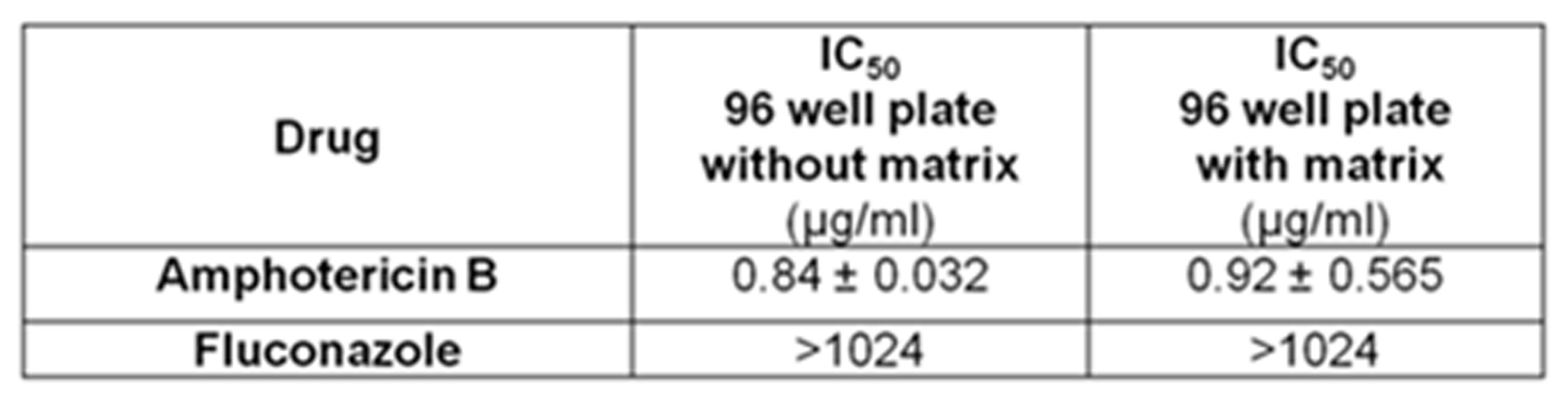

Supplement: Table S1 — IC50 in 96 well plate model. The IC50 values are calculated from the dose-response curves of fluconazole and amphotericin B against biofilms formed in a 96 well plate. (TIF) [file pone.0019036.s005.tif]
